# Pathway activity profiling of growth factor receptor network and stemness pathways differentiates metaplastic breast cancer histological subtypes

**DOI:** 10.1186/s12885-019-6052-z

**Published:** 2019-09-05

**Authors:** Jasmine A. McQuerry, David F. Jenkins, Susan E. Yost, Yuqing Zhang, Daniel Schmolze, W. Evan Johnson, Yuan Yuan, Andrea H. Bild

**Affiliations:** 1 Department of Oncological Sciences, School of Medicine, University of Utah, 2000 Circle of Hope Drive, Salt Lake City, UT 84112 USA; 2 Department of Medical Oncology and Therapeutics Research, City of Hope, 1218 S Fifth Ave, Monrovia, CA 91016 USA; 3 Division of Computational Biomedicine, School of Medicine, Boston University, 72 East Concord Street, Boston, MA 02218 USA; 4 Department of Medical Oncology and Therapeutics, City of Hope, 1500 East Duarte Road, Duarte, CA 91010 USA; 5 Department of Pathology, City of Hope, 1500 East Duarte Road, Duarte, CA 91010 USA

**Keywords:** Metaplastic breast cancer, Gene expression profiling, Survival, Invasiveness, NanoString

## Abstract

**Background:**

Gene expression profiling of rare cancers has proven challenging due to limited access to patient materials and requirement of intact, non-degraded RNA for next-generation sequencing. We customized a gene expression panel compatible with degraded RNA from formalin-fixed, paraffin-embedded (FFPE) patient cancer samples and investigated its utility in pathway activity profiling in patients with metaplastic breast cancer (MpBC).

**Methods:**

Activity of various biological pathways was profiled in samples from nineteen patients with MpBC and 8 patients with invasive ductal carcinoma with triple negative breast cancer (TNBC) phenotype using a custom gene expression-based assay of 345 genes.

**Results:**

MpBC samples of mesenchymal (chondroid and/or osteoid) histology demonstrated increased SNAI1 and BCL2L11 pathway activity compared to samples with non-mesenchymal histology. Additionally, late cornified envelope and keratinization genes were downregulated in MpBC compared to TNBC, and epithelial-to-mesenchymal transition (EMT) and collagen genes were upregulated in MpBC. Patients with high activity of an invasiveness gene expression signature, as well as high expression of the mesenchymal marker and extracellular matrix glycoprotein gene SPARC, experienced worse outcomes than those with low invasiveness activity and low SPARC expression.

**Conclusions:**

This study demonstrates the utility of gene expression profiling of metaplastic breast cancer FFPE samples with a custom counts-based assay. Gene expression patterns identified by this assay suggest that, although often histologically triple negative, patients with MpBC have distinct pathway activation compared to patients with invasive ductal TNBC. Incorporation of targeted therapies may lead to improved outcome for MpBC patients, especially in those patients expressing increased activity of invasiveness pathways.

**Electronic supplementary material:**

The online version of this article (10.1186/s12885-019-6052-z) contains supplementary material, which is available to authorized users.

## Background

Metaplastic breast cancer (MpBC) is a rare and aggressive histological subtype comprising 1 % or less of all breast cancer cases [1–3]. MpBCs are often negative for estrogen/progesterone receptor expression and HER2 amplification, yet this subtype differs in histology from invasive ductal triple negative breast cancer (TNBC) by the presence of mesenchymal (chondroid, osteoid), spindle cell, and/or squamous neoplastic cell populations [1]. Indeed, this histologically complex cancer often presents with multiple cell populations of mixed histologies. Patients with MpBC suffer from a worse outcome than those with invasive ductal TNBC, and MpBC patients demonstrate a poor response to chemotherapy [3–5]. Due to its rarity, the MpBC genome and transcriptome have only recently been studied with limited sample size [6, 7]. Comprehensive molecular profiling of MpBC and its histological subtypes is urgently needed.

**Fig. 1:**
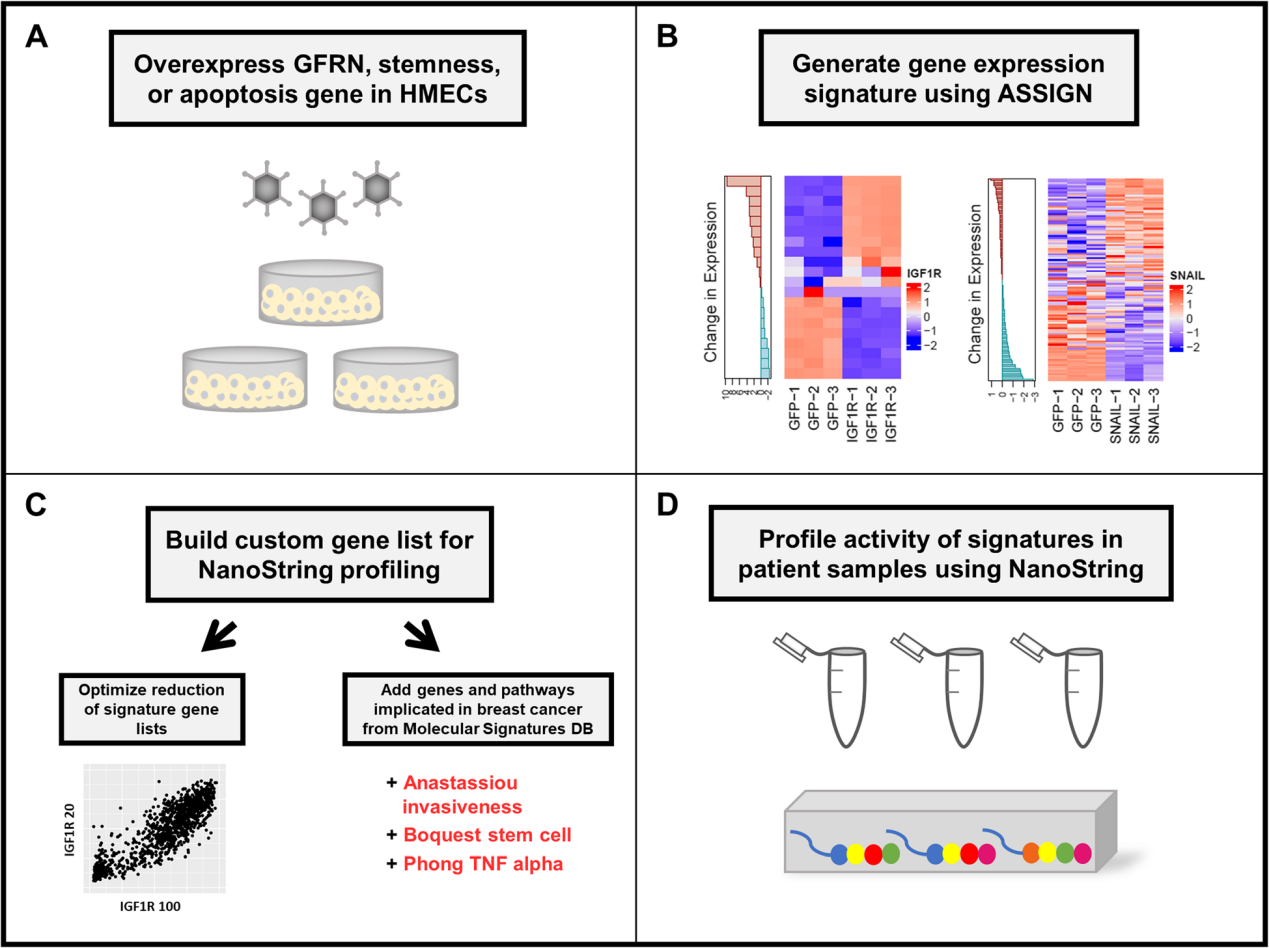
Overview of NanoString pathway activity profiling in metaplastic and triple negative breast cancer samples. **a**) Growth factor receptor network (GFRN), stemness, or apoptosis genes were individually overexpressed in normal human mammary epithelial cells (HMECs) using adenovirus delivery. **b**) The gene expression changes most correlated with induction of expression of these genes were identified. **c**) Gene lists were pared down to the fewest number of genes able to accurately predict that gene’s signature activity. These genes plus genes from other pathways relevant to breast cancer were placed on a custom NanoString panel. **d**) RNA from patient breast cancer samples was assayed using the custom NanoString panel. Figure artwork was created by the authors

Formalin-fixed, paraffin-embedded (FFPE) samples are commonly archived from breast cancer patients’ primary tumors and could prove a valuable resource with which to study MpBC omics. However, nucleic acids obtained from such samples are often degraded, thus impeding high quality profiling of transcriptomics via next generation sequencing. The NanoString nCounter platform has shown compatibility and reliability with gene expression profiling using RNA obtained from FFPE samples [8–10]. Here, we leverage the use of a custom NanoString Technologies nCounter-based assay to overcome sample degradation and to quickly and cost-effectively profile and compare pathway activity for various gene expression signatures across a set of 19 MpBC and 8 invasive ductal TNBC patient samples (Fig. 1).

## Methods

### Patient samples

Through a City of Hope IRB-approved retrospective analysis protocol, 18 FFPE and 1 fresh frozen sample from patients with MpBC, and 8 FFPE samples from patients with invasive ductal TNBC were collected for profiling (Additional file 1: Table S1). Written informed consent was obtained from all patients who participated in the study. Clinical records including demographics, treatment histories, recurrence free and overall survival, and cancer-associated mutation profiling data were reviewed and recorded for MpBC patients. MpBC samples were reviewed by a designated breast pathologist and assigned to histological subtypes including squamous, spindle cell, mesenchymal (chondroid and/or osteoid) or mixed subtype according to World Health Organization classification [11].

### Activated pathway and GFP control samples

Activated pathway or control samples were generated in normal human mammary epithelial cells (HMECs) overexpressing genes of interest or GFP, respectively, as described previously [12]. Briefly, HMECs were cultured in basal Mammary Epithelial Cell Growth Medium plus a bullet kit (MEGM, Lonza, MD, USA). HMECs were starved of bullet kit additives 36 h prior to infection with adenovirus expressing either AKT1, BAD, BCL2L11, HER2, IGF1R, RAF1, or SNAI1 for 18 h or KRAS (G12 V mutation) for 36 h at MOI of 200. Overexpression of these genes was chosen to capture core cell growth, death/survival, and stemness phenotypes.

### NanoString custom codeset

Probe gene targets for the custom gene expression panel were selected from previously published gene expression signatures (AKT1, BAD, HER2, IGF1R, KRAS G12 V, and RAF1, from Rahman et al. [12]; multi-cancer invasiveness from Anastassiou et al. [13]; stem cell signature from Boquest et al. [14]; TNF alpha signature from Phong et al. [15]) and two novel signatures (BCL2L11 and SNAI1) generated using the adenovirus infection protocol described above..

Signature gene sets from previously published AKT1, BAD, HER2, IGF1R, KRAS G12 V, and RAF1 signatures were reduced from the previously optimized RNA-sequencing-based signature lengths. Gene lists were sequentially-reduced in five gene increments down to a minimum size of five genes and each reduced gene list was used to profile cell lines from the International Cancer Benchmarking Partnership (ICBP) and breast cancer patient samples from The Cancer Genome Atlas (TCGA) using the Adaptive Signature Selection and InteGratioN toolkit (ASSIGN, [16], available from BioConductor, 10.18129/B9.bioc.ASSIGN) as described in Rahman et al. [12]. The ASSIGN pathway signature prediction scores were correlated with proteomics data for genes known to be associated with each signature as described previously [12]. Gene lists were selected to minimize the reduction of overall ASSIGN score vs. proteomics data correlation in TCGA while using a maximum of 150 genes across all six signatures (Additional file 2: Fig. S1). The reduced signature lengths for AKT1, BAD, HER2, IGF1R, KRAS G12 V, and RAF1 were 20 genes, 15 genes, 10 genes, 20 genes, 75 genes, and 50 genes, respectively.

Genes from the BCL2L11 and SNAI1 signatures were selected similarly to the method described in Rahman et al. [12]. Briefly, signature gene lists of various lengths were derived using ASSIGN to compare RNA expression from HMECs overexpressing either BCL2L11 or SNAI1 against those overexpressing GFP. For BCL2L11, candidate gene lists were subsequently used to predict pathway activity in small cell lung cancer cell lines from the Tse et al. [17] dataset (GSE10841). The BCL2L11 activity predictions from ASSIGN for these cell lines were correlated with the cell lines’ average EC50 in response to ABT-263, a Bcl-2 family inhibitor. The signature which resulted in the largest negative Spearman correlation was selected for further development. SNAI1 signature candidate gene lists were used to predict pathway activity in an immortalized normal mammary epithelial cell line (HMLE) from the Taube et al. [18] dataset (GSE24202). The signature that best separated the ASSIGN prediction scores in HMLE cells overexpressing SNAI1 from HMLE expressing empty-vector control was chosen for further development. Following selection of the BCL2L11 and SNAI1 signature gene lists, we manually screened for and removed heat shock proteins (HSP) frequently appearing in the gene lists generated by ASSIGN across pathways. Seventy-nine genes were identified as HSP genes and removed from the signatures, resulting in final signature lists containing 54 genes for BCL2L11 and 103 genes for SNAI1.

Analysis scripts for the AKT1, BAD, BCL2L11, HER2, IGF1R, KRAS G12 V, RAF1, and SNAI1 pathway signatures are available at: https://github.com/dfjenkins3/MpBC_genomics_paper.

The Anastassiou multi-cancer invasiveness, Boquest stem cell, and Phong TNF alpha signatures were reduced to 25 genes each, based on those genes with highest expression in post-treatment breast cancer patient samples profiled in Brady et al. [19]. Additional genes of interest relevant to breast cancer were also added to the panel. In total, 345 genes (336 query genes and 9 housekeeping genes) were incorporated into the custom assay (Additional file 3: Table S2).

### Patient and HMEC sample RNA extraction

RNA was extracted from patient breast cancer samples using the RNeasy FFPE kit, and from the HMEC controls using the RNeasy mini kit (both from Qiagen, CA, USA). RNA concentration was assessed with Nanodrop spectrophotometer ND-1000 and Qubit 3.0 Fluorometer (both from Thermo Scientific, CA, USA). RNA fragmentation and quality were determined by 2100 Bioanalyzer (Agilent, CA, USA).

### NanoString nCounter profiling system

The NanoString nCounter platform gene expression assay has been described previously [20]. Briefly, the NanoString nCounter platform assays gene expression directly from RNA samples via hybridization of samples with a set of multiplexed nucleotide probes. Probes for each gene target are uniquely barcoded with a series of fluorophores. Fluorescence microscopy imaging of sample-hybridized fluorophore-labeled probes generates quantitative counts data for each gene in each sample.

For gene expression profiling on the nCounter system, patient sample or HMEC control RNA was first hybridized with the custom 345-gene codeset (NanoString Technologies, WA, USA) at 65 °C for 16 h. Post-hybridization probe:target mixture was then purified and quantified via nCounter MAX Digital Analyzer (NanoString Technologies, WA, USA).

### Pathway activity profiling in patient samples

Raw NanoString counts data were normalized to internal positive control probes and housekeeping genes using nSolver Software (NanoString Technologies, WA, USA) version 4.0, according to default parameters, except for background threshold count value was set to 20. Pathway probabilities for AKT1, BAD, BCL2L11, KRAS G12 V, HER2, IGF1R, RAF1, and SNAI1 signatures were calculated using ASSIGN, according to the same parameters as in Rahman et al. [12], with adaptive signature selection set to false. Pathway scores for Anastassiou multi-cancer invasiveness, Phong TNF alpha, and Boquest stem cell signatures were calculated using ASSIGN as above, with adaptive signature selection set to true.

### Differential gene expression and biological pathway enrichment analysis

Differential gene expression analysis was performed using the NanoStringDiff package, version 1.10.0 for R (available from BioConductor, 10.18129/B9.bioc.NanoStringDiff) using default settings [21]. This package uses a negative binomial-based model appropriate for discrete counts data, and employs a normalization step incorporating data from the internal nCounter positive and negative controls and the panel housekeeping controls to identify differentially-expressed genes across groups. The package adjusts for false discovery using the Benjamini-Hochberg method. Genes passing the q < 0.05 false discovery cutoff were considered for pathway enrichment analysis using Ingenuity Pathway Analysis (IPA) software (Qiagen Silicon Valley, CA, USA). Analyses in IPA were run with the “reference set” parameter set to the input list of genes assayed on the NanoString panel to account for sampling bias of genes chosen for the panel. IPA uses a right-tailed Fisher’s exact test to calculate the probability that genes belonging to particular biological pathways from its curated knowledge base are enriched in input datasets due to chance. IPA canonical pathways with *p* < 0.05 are reported herein.

### Statistics

Statistical tests were performed using Prism version 6.0 (GraphPad, CA, USA). Comparison of ASSIGN pathway activity scores across groups was performed using one-way ANOVA followed by Tukey’s post hoc test. Survival analyses were performed using the Kaplan-Meier log-rank method, with hazard ratios (HR) and 95% confidence intervals (CI) reported. For survival analyses, patients were grouped by median pathway activity score and the sample with median value was included in the group containing its closest numerical value. The single sample with mesenchymal histology was grouped with samples with mixed mesenchymal and spindle cell histology for analyses.

## Results

### Patient cohort characteristics

A total of 19 cases of MpBC from 1996 to 2014 were included. The median patient age at diagnosis was 68 years (range: 35–86 years). A diverse range of histological subtypes was represented in the patient cohort, including 32% (6/19) squamous, 37% (7/19) spindle cell, 16% (3/19) mixed squamous and spindle cell, 10% (2/19) mixed spindle cell and mesenchymal, and 5% (1/19) mesenchymal samples (Table 1). Representative hematoxylin and eosin stained slides demonstrating histology of each subtype can be found in Additional file 4: Fig. S2. The majority of patients’ cancers were categorized as ER−/PR−/HER2-, with 2 patients’ HER2 status unknown. Median follow-up time for all 19 patients was 64 months (range: 5–220) and for those patients alive at time of analysis, 84 months (range: 64–220).

**Table 1:** Cohort characteristics for 19 patients with metaplastic breast cancer

Patient Characteristics	*n* = 19	%
Age:	Median: 68	Range: 35–86
< 50	3	15.8
50 to < 70	7	36.8
≥ 70	9	47.4
Race:		
Asian	2	10.5
Hispanic	3	15.8
Non-Hispanic White	14	73.7
Breast Cancer Stage:		
I	2	10.5
II	15	78.9
III	1	5.3
IV	1	5.3
ER, PR, HER2 Status:		
ER-, PR-, HER2-	17	89.5
ER-, PR-, HER2 unknown	2	10.5
Histological Subtype:		
Mesenchymal	1	5.3
Mixed spindle cell and mesenchymal	2	10.5
Mixed squamous and spindle cell	3	15.8
Spindle cell	7	36.8
Squamous	6	31.6

### Performance of RNA-seq based signatures on NanoString platform

**Fig. 2:**
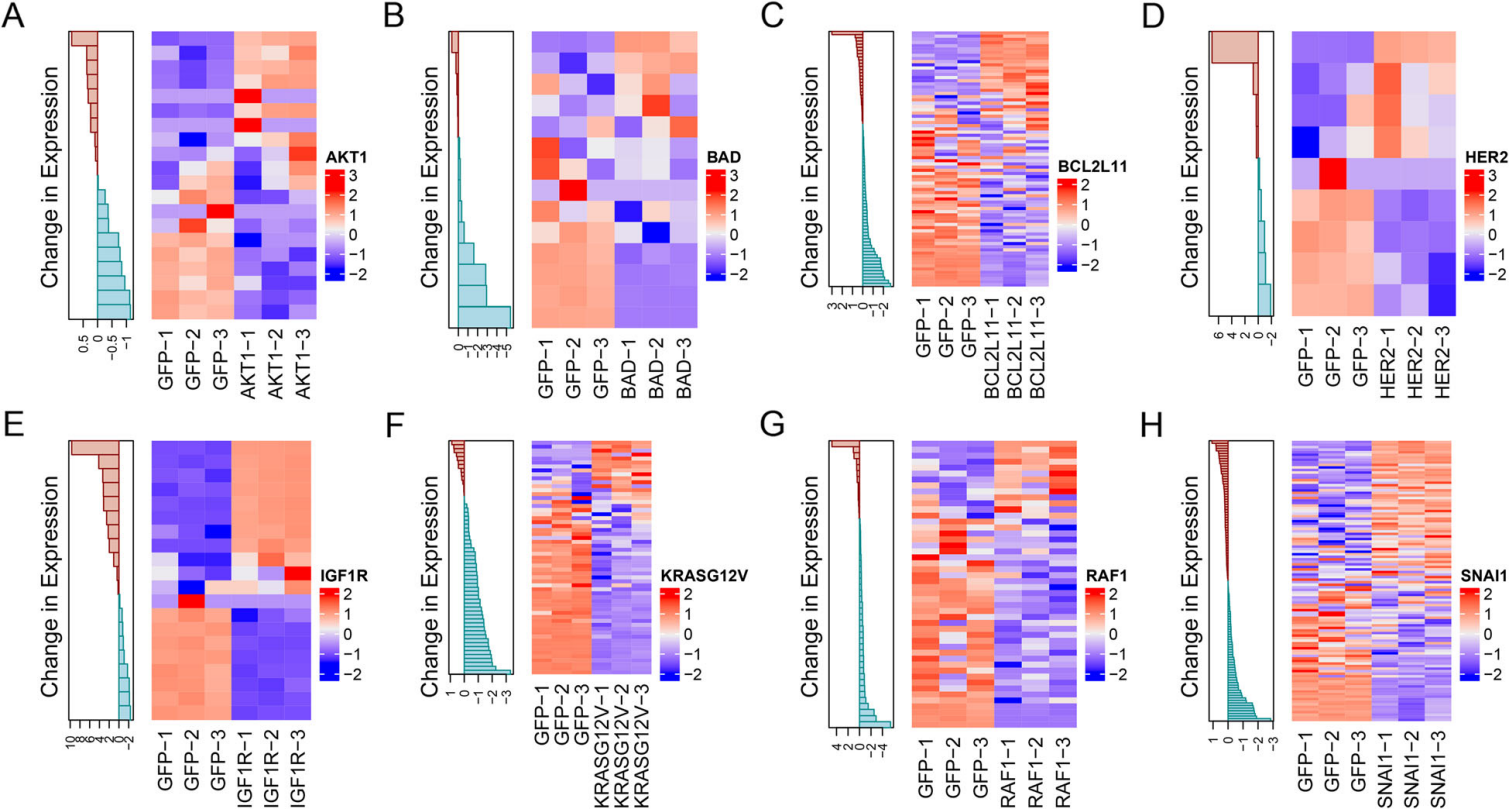
Gene expression changes are captured via a custom NanoString panel. Overexpression of **a**) AKT1 **b**) BAD **c**) BCL2L11 **d**) HER2 **e**) IGF1R **f**) KRAS G12 V **g**) RAF1 and **h**) SNAI1 genes led to distinct downstream changes in gene expression

We converted gene expression signatures originally created using RNA-sequencing data for use with the NanoString gene expression profiling platform. To re-optimize the signatures to best capture pathway activity via NanoString, RNA from control HMEC samples overexpressing each gene of interest and from HMECs overexpressing GFP was assayed on the NanoString platform using the custom codeset, and the top gene expression changes between groups were identified using ASSIGN (Fig. 2). These changes in gene expression identified in the control samples were then used to profile pathway activity in patient samples.

### Metaplastic breast cancer histological subtypes demonstrate differential pathway activation

**Fig. 3:**
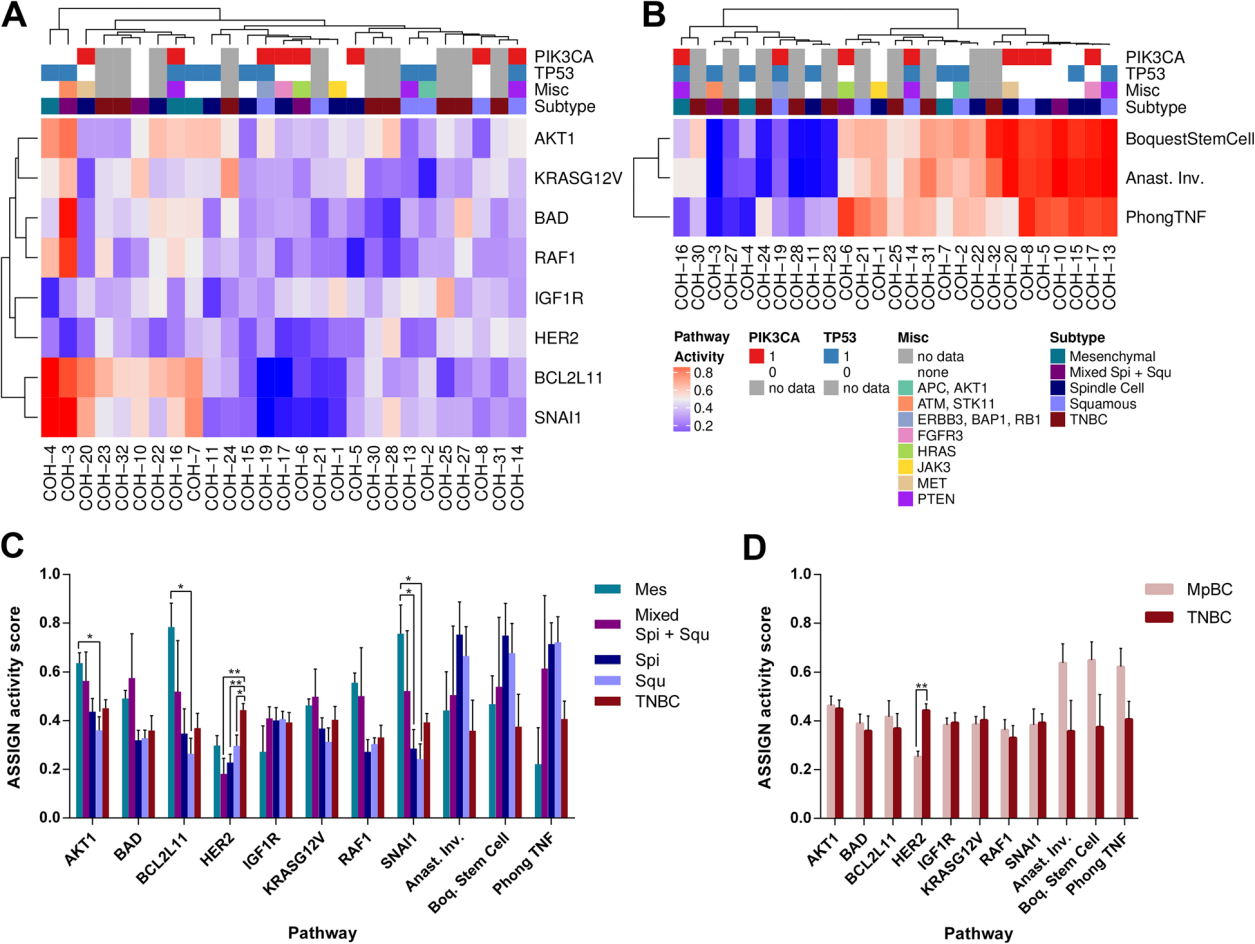
Pathway activity by metaplastic subtype. **a**) Heatmap of ASSIGN pathway probabilities for metaplastic and triple negative breast cancer samples for experimentally-derived signatures and **b**) Heatmap of ASSIGN pathway probabilities for metaplastic and triple negative breast cancer samples for literature-derived signatures. TP53, PIK3CA, and “other” boxes indicate presence or absence of clinically significant mutations identified via Onco48 or Foundation Medicine panel. **c**) ASSIGN pathway activity scores by histological cancer subtype and **d**) ASSIGN pathway activity scores in all metaplastic and triple negative samples. *p < 0.05; **p < 0.01 via post-hoc Tukey test. Bars represent standard error of the mean. Mes: mesenchymal, spi: spindle cell, squ: squamous, spi + squ: mixed spindle cell and squamous, Anast. Inv.: Anastassiou multi-cancer invasiveness. Boq.: Boquest

Unsupervised hierarchical clustering of pathway activity scores for growth factor receptor network (GFRN), stemness, and apoptosis pathways revealed several broad clusters of pathway activity across MpBC and TNBC patients (Fig. 3a-b). Notably, MpBC and TNBC patient samples did not cluster exclusively; rather, these samples were interleaved across clusters. Further, MpBC patient samples did not group distinctly by subtype; however, patient samples with a mesenchymal cell population (chondroid and/or osteoid) grouped in high SNAI1/BCL2L11 pathway activity clades (left side of heatmap; Fig. 3a), while all uniformly squamous samples grouped in low SNAI1/BCL2L11 pathway activity clades (right side of heatmap, Fig. 3a). Indeed, samples with any mesenchymal cell population had significantly higher SNAI1 pathway activity scores than patients of the spindle and squamous subtypes (ANOVA, *p* = 0.0131; Fig. 3c). Similarly, mesenchymal samples demonstrated significantly increased BCL2L11 and marginally significantly increased AKT1 pathway activity compared to squamous patients (BCL2L11: ANOVA, *p* = 0.0337; AKT1: ANOVA, *p* = 0.0542, Fig. 3c).

Interestingly, HER2 pathway activity was significantly higher in TNBC samples than in MpBC samples (Student’s t-test, *p* < 0.001; Fig. 3d). Specifically, spindle cell, squamous and mixed spindle/squamous subtype samples had significantly lower HER2 pathway activity than TNBC samples (ANOVA, *p* < 0.001; Fig. 3c). All patient MpBC and TNBC samples were clinically categorized as negative for HER2 amplification or HER2 status unknown; however, all samples expressed *ERBB2*, with TNBC samples demonstrating significantly increased *ERBB2* expression compared to MpBC samples (Additional file 5: Fig. S3). Differences in expression of the other 9 genes in the HER2 gene expression signature also contributed to differential pathway activity between MpBC and TNBC samples. No differences were seen in pathway activity across subtypes for the other pathways profiled, including BAD, KRASG12 V, IGF1R, RAF1, Anastassiou invasiveness, Boquest stem cell and Phong TNF alpha (Fig. 3c-d).

### Differences in gene expression across subtypes

We examined gene expression differences across the panel of NanoString genes using NanoStringDiff, an R package designed to identify gene expression differences from the discrete counts data generated by the NanoString platform [21]. Gene expression profiling revealed differences between MpBC and TNBC samples as well as between samples of different MpBC histological subtypes. Fifty-seven genes were differentially expressed between MpBC and TNBC samples (Table 2). Genes down-regulated in MpBC included, among others, *CD24*, keratinocyte-related genes such as *CALML5* and *KRT81* and late cornified envelope genes, *LCE1F*, *LCE3D*, and *LCE3E*, which were largely not expressed in MpBC samples, but were expressed in the majority of TNBC samples. Genes up-regulated in MpBC included cytokine genes *IL6* and *IL8*, EMT-related genes *FN1* and *CTGF*, and genes involved in extracellular matrix synthesis and adhesion: *COL1A1*, *COL5A1*, *COL5A2*, *ICAM1*, and *HAS2* (Table 2).

**Table 2:** Genes differentially expressed between metaplastic breast cancer and invasive ductal triple negative breast cancer samples

Gene	Log_2_ Fold Change	*p*-value	FDR-adjusted *p*-value
Genes upregulated in metaplastic samples			
PTHLH	4.11	1.90E-05	4.43E-04
IGFBP3	1.97	6.59E-05	1.23E-03
ICAM1	1.71	6.57E-04	8.83E-03
IER3	1.72	8.62E-04	1.07E-02
BST2	1.56	1.23E-03	1.43E-02
HAS2	1.93	2.48E-03	2.45E-02
PDPN	1.95	2.47E-03	2.45E-02
IL6	2.31	2.64E-03	2.54E-02
COL5A1	1.52	3.86E-03	3.12E-02
FN1	1.53	3.59E-03	3.12E-02
IL8	2.02	4.87E-03	3.80E-02
PLAT	1.77	5.52E-03	3.99E-02
COL5A2	1.52	5.77E-03	4.04E-02
COL1A1	1.67	5.93E-03	4.07E-02
INHBA	1.49	6.65E-03	4.47E-02
CDH11	1.15	7.22E-03	4.53E-02
MYLK	1.32	7.13E-03	4.53E-02
CTGF	1.37	7.31E-03	4.53E-02
Genes downregulated in metaplastic samples			
PRR9	−5.10	2.55E-15	8.58E-13
LCE3E	−31.84	3.33E-14	5.60E-12
LCE1F	−5.76	1.43E-11	1.60E-09
FAM25A	−3.87	2.89E-10	2.43E-08
AGPAT9	−2.65	5.29E-10	3.45E-08
DUSP8	−2.14	6.17E-10	3.45E-08
FAM83A-AS1	−3.61	4.15E-08	1.99E-06
FGFBP2	−0.26	1.20E-07	5.06E-06
CHAC1	−2.84	3.57E-07	1.33E-05
HSPE1	− 2.07	1.10E-06	3.69E-05
CD24	−2.90	1.57E-06	4.80E-05
ALOXE3	−3.54	4.81E-06	1.35E-04
ERBB2	−1.24	1.22E-05	3.16E-04
RIMS3	−1.36	1.98E-05	4.43E-04
ACTBL2	−3.04	2.11E-05	4.44E-04
KLK6	−3.51	3.69E-05	7.30E-04
LCE3D	−4.12	8.55E-05	1.51E-03
ABCB1	−2.20	1.15E-04	1.94E-03
LOC100130238	−2.86	2.12E-04	3.39E-03
BMP6	−2.06	4.06E-04	6.19E-03
TMCC2	−1.55	5.38E-04	7.86E-03
LEP	−2.77	6.05E-04	8.47E-03
ZSCAN12P1	−1.70	8.46E-04	1.07E-02
CCL26	−1.96	9.29E-04	1.11E-02
MAL	−2.26	1.38E-03	1.54E-02
ERBB3	−1.85	2.29E-03	2.41E-02
NOV	−1.52	2.26E-03	2.41E-02
PAPL	−1.91	2.88E-03	2.69E-02
DSCAM	−2.87	3.76E-03	3.12E-02
KRT81	−2.34	3.69E-03	3.12E-02
TAGLN3	− 2.02	3.90E-03	3.12E-02
ZNF165	−1.39	3.44E-03	3.12E-02
CALML5	−2.55	5.00E-03	3.82E-02
DIRAS3	−1.69	5.52E-03	3.99E-02
ARHGAP33	−0.66	5.58E-03	3.99E-02
RASD2	−1.24	6.92E-03	4.53E-02
FAM46B	−1.20	7.41E-03	4.53E-02
PDGFB	−0.79	8.17E-03	4.90E-02

*FDR* false-discovery rate

Further, to explore subtype-specific gene expression, we identified genes differentially expressed in each MpBC subtype. Twenty-four panel genes were significantly differentially expressed between spindle cell MpBCs and all other MpBCs (Benjamini-Hochberg adjusted *p* < 0.05, Table 3). Squamous subtype samples had 36 differentially expressed genes and mesenchymal subtype samples had 24 genes differentially expressed compared to all other MpBC samples (Table 3).

**Table 3:** Genes differentially expressed between metaplastic breast cancer subtypes

Gene	Log_2_ Fold Change	*p*-value	FDR-adjusted *p*-value
Genes differentially expressed in Mesenchymal vs. all other MpBC types			
AQP5	10.36	< 2.22E-16	< 2.22E-16
IL1A	−3.83	1.36E-11	2.28E-09
EXTL1	9.07	6.84E-11	7.67E-09
TNFRSF11B	5.02	1.36E-09	1.14E-07
FGFBP2	6.91	2.22E-08	1.49E-06
PSG5	−6.16	2.84E-08	1.59E-06
CXCL5	−2.49	1.34E-07	6.43E-06
CA6	21.62	2.11E-05	8.85E-04
CCL2	−3.10	2.39E-05	8.92E-04
IRF1	−2.48	8.46E-05	2.84E-03
IFI35	−2.20	1.23E-04	3.76E-03
STAT1	−1.98	4.09E-04	1.15E-02
TYMP	−2.00	5.00E-04	1.29E-02
EPSTI1	−2.51	6.65E-04	1.40E-02
ICAM1	−2.42	6.29E-04	1.40E-02
OAS3	−1.65	6.67E-04	1.40E-02
BST2	−2.30	7.25E-04	1.43E-02
ARC	2.43	9.32E-04	1.74E-02
TAP1	−2.19	1.02E-03	1.81E-02
SAMHD1	−1.76	2.34E-03	3.93E-02
SYNGR1	1.30	2.82E-03	4.31E-02
TNFRSF10B	−1.47	2.76E-03	4.31E-02
CXCL11	−2.64	3.29E-03	4.60E-02
STC1	−2.23	3.21E-03	4.60E-02
Genes differentially expressed in Spindle cell vs. all other MpBC types			
SPRR1A	−5.30	6.48E-13	2.18E-10
SPRR2A	−3.67	3.28E-12	5.51E-10
SPRR2D	−22.62	8.87E-12	9.94E-10
AQP5	5.73	3.26E-11	2.74E-09
EXTL1	2.98	6.86E-11	4.61E-09
CXCL6	6.08	2.27E-09	1.27E-07
LY6D	−1.56	2.75E-09	1.32E-07
KRT4	−2.64	9.36E-09	3.93E-07
TFPI2	2.98	1.17E-08	4.37E-07
CXCL5	3.10	1.61E-07	5.41E-06
NEFM	5.58	2.92E-05	8.92E-04
IL1B	2.65	7.61E-05	2.13E-03
MAOB	2.57	2.80E-04	7.25E-03
FN1	1.66	4.80E-04	1.04E-02
CCL2	1.53	4.94E-04	1.04E-02
LEP	−21.29	4.51E-04	1.04E-02
MMP9	2.92	5.87E-04	1.16E-02
CXCL3	2.07	9.76E-04	1.64E-02
HSPA6	1.31	9.46E-04	1.64E-02
VIPR1	−2.30	9.44E-04	1.64E-02
ROR1	1.08	2.21E-03	3.53E-02
OXTR	1.66	2.39E-03	3.65E-02
TNFRSF10D	1.62	2.60E-03	3.80E-02
DKK1	2.42	3.30E-03	4.62E-02
Genes differentially expressed in Squamous vs. all other MpBC types			
AQP5	−24.83	< 2.22E-16	< 2.22E-16
ELF5	−2.79	< 2.22E-16	< 2.22E-16
SPRR1A	6.65	3.37E-12	3.77E-10
SPRR2A	6.30	4.54E-12	3.81E-10
TF	−3.00	1.90E-11	1.28E-09
HSP90AA4P	−2.99	4.67E-11	2.62E-09
CA6	−8.88	1.43E-10	6.87E-09
EXTL1	−19.35	2.62E-10	1.10E-08
PSG5	0.20	1.83E-09	6.84E-08
GLYATL2	3.00	7.10E-09	2.39E-07
PI3	5.49	7.14E-08	2.18E-06
TCN1	3.97	3.25E-05	8.59E-04
EPGN	2.68	3.32E-05	8.59E-04
DHRS9	2.21	7.43E-05	1.78E-03
SPRR2D	11.54	1.09E-04	2.44E-03
SLC47A2	4.33	1.52E-04	3.19E-03
DSCAM	−23.49	1.79E-04	3.53E-03
ID4	−2.93	2.20E-04	4.11E-03
ALDH3B2	3.18	2.89E-04	5.12E-03
CITED1	3.80	3.23E-04	5.43E-03
EEF1A2	3.03	4.19E-04	6.40E-03
CCL20	2.22	4.13E-04	6.40E-03
C12orf39	2.70	5.40E-04	7.89E-03
STEAP4	2.28	6.83E-04	9.56E-03
PPL	2.03	7.28E-04	9.79E-03
NEFM	−5.96	9.26E-04	1.20E-02
HAS2	−2.14	1.18E-03	1.47E-02
LCE3D	4.05	1.26E-03	1.52E-02
CDRT1	2.92	1.60E-03	1.85E-02
PTHLH	2.48	1.74E-03	1.88E-02
PRSS22	1.99	1.72E-03	1.88E-02
ALDH1A1	1.67	2.01E-03	2.11E-02
TNFAIP2	1.41	2.30E-03	2.35E-02
LEP	8.40	2.94E-03	2.91E-02
ELF3	2.34	4.25E-03	4.08E-02
GRHL3	2.08	4.82E-03	4.50E-02

*MpBC* metaplastic breast cancer. *FDR*: false discovery rate

Next, we interrogated non-GFRN pathway dysregulation at the subtype level by assessing the differentially expressed genes identified by NanoStringDiff for enrichment of genes belonging to the same pathway in the canonical pathways database curated by IPA. Genes differentially expressed between MpBC and TNBC samples were enriched for genes in the hepatic fibrosis and atherosclerosis pathways (Table 4). Differentially expressed genes from the mesenchymal subtype were enriched for interferon signaling, IL-17 signaling, (a) granulocyte adhesion, and helper T cell differentiation pathway members. Similarly, IL-17 signaling and (a) granulocyte adhesion pathways were identified as enriched in spindle cell differentially expressed genes, as several genes up-regulated in mesenchymal samples were down-regulated in spindle cell samples. No pathways were significantly enriched in genes differentially expressed in squamous subtype samples.

**Table 4:** Canonical pathways enriched in genes differentially expressed between subtypes

Pathway	*p*-value	Overlap (%)	Overlap (gene number)
MpBC vs. TNBC			
Hepatic fibrosis /Hepatic stellate cell activation	1.80E-03	39.3	11/28
Atherosclerosis signaling	2.13E-02	40	6/15
Mesenchymal vs. other MpBC			
Interferon signaling	6.83E-04	57.1	4/7
Granulocyte adhesion and diapedesis	1.18E-03	31.6	6/19
Agranulocyte adhesion and diapedesis	4.74E-03	29.4	5/17
Type I Diabetes Mellitus signaling	1.01E-02	42.9	3/7
Th1 pathway	1.01E-02	42.9	3/7
Differential regulation of cytokine production in intestinal epithelial cells by IL-17A and IL-17F	1.48E-02	66.7	2/3
Th1 and Th2 activation pathway	1.53E-02	37.5	3/8
HMGB1 signaling	1.77E-02	26.7	4/15
Dendritic cell maturation	1.77E-02	26.7	4/15
IL-17 signaling	2.19E-02	33.3	3/9
iNOS signaling	2.83E-02	50.0	2/4
T helper cell differentiation	2.83E-02	50.0	2/4
Retinoic acid mediated apoptosis signaling	2.83E-02	50.0	2/4
IL-17A signaling in fibroblasts	2.83E-02	50.0	2/4
IL-15 production	2.83E-02	50.0	2/4
Production of nitric oxide and reactive oxygen species in macrophages	2.97E-02	30.0	3/10
LXR/RXR activation	2.97E-02	30.0	3/10
Hepatic fibrosis /Hepatic stellate cell activation	4.28E-02	17.9	5/28
Spindle vs. other MpBC			
Agranulocyte adhesion and diapedesis	5.60E-05	41.2	7/17
Granulocyte adhesion and diapedesis	1.18E-03	31.6	6/19
Role of IL-17F in allergic inflammatory airway diseases	1.30E-03	50.0	4/8
Role of IL-17A in Arthritis	1.30E-03	50.0	4/8
Glucocorticoid receptor signaling	2.13E-03	28.6	6/21
Osteoarthritis pathway	4.74E-03	29.4	5/17
Role of IL-17A in Psoriasis	6.04E-03	50.0	3/6
Hepatic fibrosis /Hepatic stellate cell activation	1.04E-02	21.4	6/28
Differential regulation of cytokine production in macrophages and T helper cells by IL-17A and IL-17F	1.48E-02	66.7	2/3
Differential regulation of cytokine production in intestinal epithelial cells by IL-17A and IL-17F	1.48E-02	66.7	2/3
Airway pathology in Chronic Obstructive Pulmonary Disease	1.48E-02	66.7	2/3
IL-17A Signaling in airway cells	1.53E-02	37.5	3/8
IL-17A signaling in fibroblasts	2.83E-02	50.0	2/4
LXR/RXR activation	2.97E-02	30.0	3/10
TREM1 signaling	3.89E-02	27.3	3/11
Squamous vs. other MpBC			
No significant pathways to report			

*MpBC* metaplastic breast cancer, *TNBC* invasive ductal carcinoma of triple negative breast cancer phenotype

### Invasiveness markers and patient survival

**Fig. 4:**
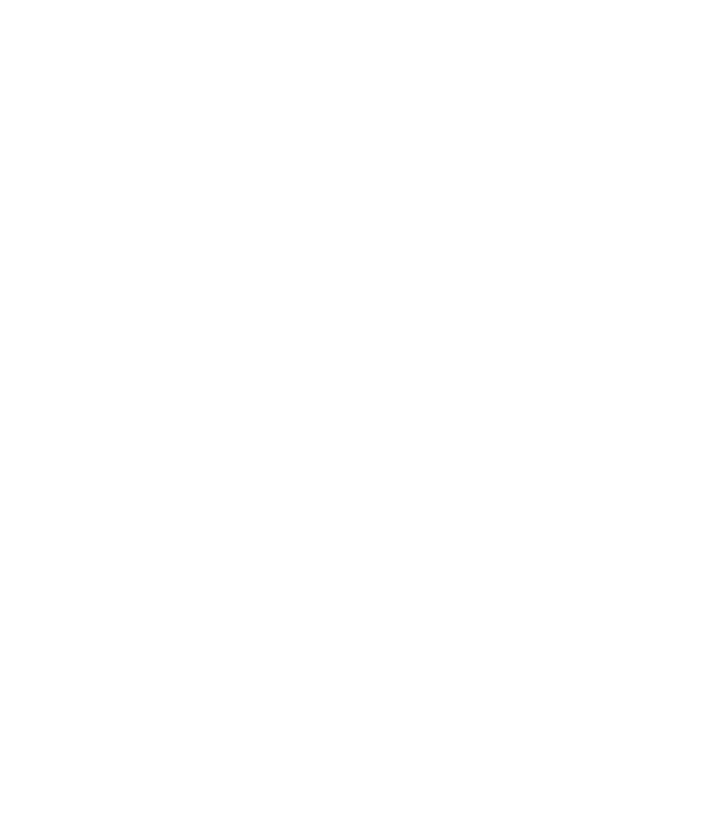
Patient survival correlates with epithelial-to-mesenchymal transition and invasiveness markers. Patients with **a**) high Anastassiou invasiveness signature activity, **b**) high SPARC gene expression and **c**) low KRAS G12 V pathway activity experience a worse outcome

To examine the relationship between pathway activity and survival, we stratified patients by median ASSIGN pathway activity score for all pathways assayed, and assessed patient recurrence-free survival (RFS) and overall survival (OS) within each group. Patients with above-median Anastassiou invasiveness pathway activity experienced shorter RFS and OS than those with equal to or below-median pathway activity (RFS: *p* = 0.021, HR = 5.82, 95% CI = 1.31–25.84; OS *p* = 0.02, HR = 5.77, 95% CI = 1.32–25.24; Fig. 4a). Patients with below-median KRAS G12 V pathway activity experienced a worse outcome compared to those patients with equal-to or above-median KRAS G12 V pathway activity (RFS: *p* = 0.0145, HR = 6.55, 95% CI = 1.45–29.55; OS: *p* < 0.001, HR = 14.14, CI = 3.10–64.40; Fig. 4c). There was no significant difference in outcome identified between patients stratified by median pathway activity for the remaining pathways assessed with the NanoString panel.

Previous studies have identified that expression of mesenchymal markers including SPARC, VIM, and TWIST negatively correlate with MpBC patient survival [22, 23]. In the present study, patients with above-median SPARC expression experienced shorter recurrence-free and overall survival times than patients with equal-to or below-median SPARC expression (RFS *p* = 0.023, HR = 5.52, 95% CI = 1.26–24.1; OS *p* = 0.023, HR = 5.41, 95% CI = 1.26–23.2). Conversely, patients bifurcated by median VIM expression or by median SNAI1 pathway activity did not experience differences in outcome (Additional file 6: Fig. S4).

## Discussion

Elucidation of the omics underlying rare cancer types such as MpBC requires methods to accurately profile limited samples available from these cancers. Our results demonstrate the utility of RNA collected from FFPE samples and profiled with the NanoString platform to obtain interpretable gene expression and pathway activity data for patients with MpBC. Using this platform, we identified differences in gene expression and pathway activity between MpBC and invasive ductal TNBC samples, as well as between samples from different MpBC subtypes.

Several genes with potential implications on patient treatment were found significantly differentially expressed between MpBC and TNBC samples. One such gene, *CD24*, was down-regulated in MpBC. Interestingly, low expression or lack of expression of the CD24 protein has long been considered a marker of breast cancer stem cells, and various clinical trials are underway to target the cancer stem cell population in breast cancer [24–26]. Additionally, we identified *COL1A1* up-regulation in MpBC samples. The protein product of the *COL1A1* gene forms part of the type I collagen protein complex, which has previously been identified as up-regulated in mesenchymal MpBCs when compared to adjacent normal tissue [27]. Further, high expression of the *COL1A1* gene and protein has been associated with shorter recurrence free and overall survival in breast cancer, as well as with response to cisplatin [28, 29]. Additionally, we identified increased *HAS2* in MpBC samples. A previous study found expression of this enzyme involved in hyaluronan synthesis in 72.7% of patients with MpBC, compared to only 56% of patients with invasive ductal TNBC, and 25.2% of patients with invasive ductal carcinoma of ER, PR, or HER2-positive phenotypes [30]. Clinical trials investigating treatment of patients with high hyaluronan levels with recombinant hyaluronidase are currently underway in multiple cancer types [31–33].

At the pathway activity level, profiling results demonstrated increased BCL2L11, SNAI1, and AKT1 pathway activity in patient samples with a histologic mesenchymal (chondroid or osteoid) component. This finding supports that of Gwin et al. [34], who identified increased SNAI1 gene expression in chondroid MpBC tumors, and that of Taube et al. [18], who found high SNAI1 expression in a set of 12 metaplastic patient samples. Based on these findings, inhibition of SNAI1 pathway components may be a viable strategy for improving outcomes for patients with mesenchymal MpBC. While there are currently no FDA-approved SNAI1 inhibitors, the histone deacetylase (HDAC) inhibitors panobinostat and entinostat have been shown to reduce expression of SNAI1 and other EMT markers [35–37]. HDAC inhibitors are currently FDA-approved for use in some cancers, and thus may be an implementable strategy for treatment of MpBC tumors with high SNAI1 activity.

Similarly, we identified increased BCL2L11 pathway activity in patients with mesenchymal MpBC. Increased SNAI2-driven *BCL2L11*-encoded protein BIM expression was identified by Merino et al. [38] at the proliferating edge of two metaplastic breast cancer patient-derived xenografts, and it was speculated that this expression may play a role in tumor cell dissemination and metastasis. This same leading-edge expression of BIM was not present in TNBC and ER+ xenografts. Future experiments are needed to clarify the role of increased BIM in MpBC tumors, and to determine whether modulation of MAPK pathway activity upstream of BIM improves outcomes for patients with mesenchymal MpBC.

In the present cohort, patient samples with high Anastassiou invasiveness pathway activity and high expression of the extracellular matrix glycoprotein SPARC experienced worse outcomes. SPARC expression has been associated with invasiveness phenotype in patients with ductal carcinoma in situ, as well as with poor survival in patients with TNBC [39, 40]. Thus, a treatment strategy capable of reducing the invasiveness potential of metaplastic cancer cells may benefit MpBC patient outcome. Lack of KRAS activity to drive poor outcome in the present patient cohort may reflect the extent to which aggressive MpBCs are driven by stemness/invasiveness pathways not related to MAPK pathway activity.

MpBC tumors are notorious for their failure to respond to chemotherapy; however, chemotherapy remains the standard of care for TNBC, including triple-negative MpBC [5, 41]. Thus, identification of targetable pathways altered in MpBC is necessary for improving patient outcomes. Multiple ongoing trials including ARTEMIS and I-SPY2 are testing a precision medicine approach for TNBC treatment [42–44]. Patients with MpBC may similarly benefit from a precision medicine approach, which may be further tailored to the patient’s specific MpBC subtype. Such an approach might leverage tumor transcriptomic profiling at time of patient diagnosis to determine if MpBC patients would benefit from specific targeted therapies.

MpBC is a remarkably rare cancer, and it is important to note the limitations in our conclusions due to the limited sample size from a single institution. However, data from the current study corroborate findings from other MpBC studies published to date. One such study examined gene expression differences across MpBC subtypes via RNA sequencing [6]. As in the present study, Piscuoglio et al. [6] also identified genes *ALDH3B2*, *CDRT1*, *ELF3*, *EXTL1*, *GLYATL2*, *PI3*, *PPL*, and *PRSS22* as differentially expressed in the squamous subtype and genes *AQP5*, *EXTL1*, *MMP9*, *NEFM*, and *VIPR1* in the spindle subtype. Further, our identification of increased *IL8*, *IL6*, *HAS2*, and *ICAM1*, as well as decreased *ERBB2* in MpBC samples matches findings from a microarray comparison of gene expression between metaplastic breast cancers and ductal carcinomas of the breast [22]. At the pathway activity level, high SNAI1 activity and increased expression of stemness and EMT markers have been identified in the present cohort as well as in other MpBC patient cohorts [18, 34].

## Conclusions

This study demonstrates the utility of applying a pathway-specific multiplex gene expression assay in profiling FFPE-derived RNA from patients with MpBC. Gene expression profiling across different MpBC tumor subtypes showed coordinate dysregulation of growth and survival pathways that was distinct from immune and stemness cell states. Further, RAS signaling activity and activity of pathways related to cancer invasiveness were associated with patient outcome in this cancer type. Future studies to validate findings in a larger MpBC patient cohort are warranted.

## Additional files

Additional file 1 **Table S1**. Breast cancer patient sample characteristics. MpBC: metaplastic breast cancer. TNBC: triple negative breast cancer. FFPE: formalin-fixed paraffin-embedded. (XLSX 10 kb)

Additional file 2 **Fig. S1**. Reduction of RNA-sequencing based gene expression signature gene lists for NanoString panel in a) ICBP and b) TCGA for AKT1, BAD, HER2, IGF1R, KRASG12 V, and RAF1 pathways. (PNG 2175 kb) (PNG 415 kb)

Additional file 3 **Table S2**. Custom NanoString panel gene list. (PNG 415 kb) (XLSX 19 kb)

Additional file 4 **Fig. S2**. Representative hematoxylin and eosin slides for a) squamous b) spindle cell c) mesenchymal (chondroid) and d) mesenchymal (osteoid) metaplastic breast cancer histological subtypes. (TIF 574 kb) (PNG 2175 kb)

Additional file 5 **Fig. S3**. ERBB2/HER2 gene expression in MpBC and TNBC samples. (XLSX 19 kb) (TIF 272 kb)

Additional file 6 **Fig. S4**. a) Increased VIM expression or b) SNAI1 pathway activity do not associate with worse outcome. (TIF 272 kb) (TIF 574 kb)

## Data Availability

The datasets and code generated as part of the current study are available in the GitHub repository, at the following link: https://github.com/dfjenkins3/MpBC_genomics_paper.

## Abbreviations

ASSIGN: Adaptive Signature Selection and InteGratioN toolkit
CI: Confidence interval
EMT: Epithelial-to-mesenchymal transition
FFPE: Formalin-fixed, paraffin-embedded
GFRN: Growth factor receptor network
HDAC: Histone deacetylase
HMEC: Human mammary epithelial cell
HR: Hazard ratio
HSP: Heat shock protein
ICBP: International Cancer Benchmarking Partnership
IPA: Ingenuity Pathway Analysis
MpBC: Metaplastic breast cancer
OS: Overall survival
RFS: Recurrence-free survival
TCGA: The Cancer Genome Atlas
TNBC: Triple negative breast cancer
