# Supplementary figures and images for: Pathway activity profiling of growth factor receptor network and stemness pathways differentiates metaplastic breast cancer histological subtypes
Source: BMC Cancer. 2019 Sep 5;19:881. doi: 10.1186/s12885-019-6052-z (PMC6727561; doi:10.1186/s12885-019-6052-z)

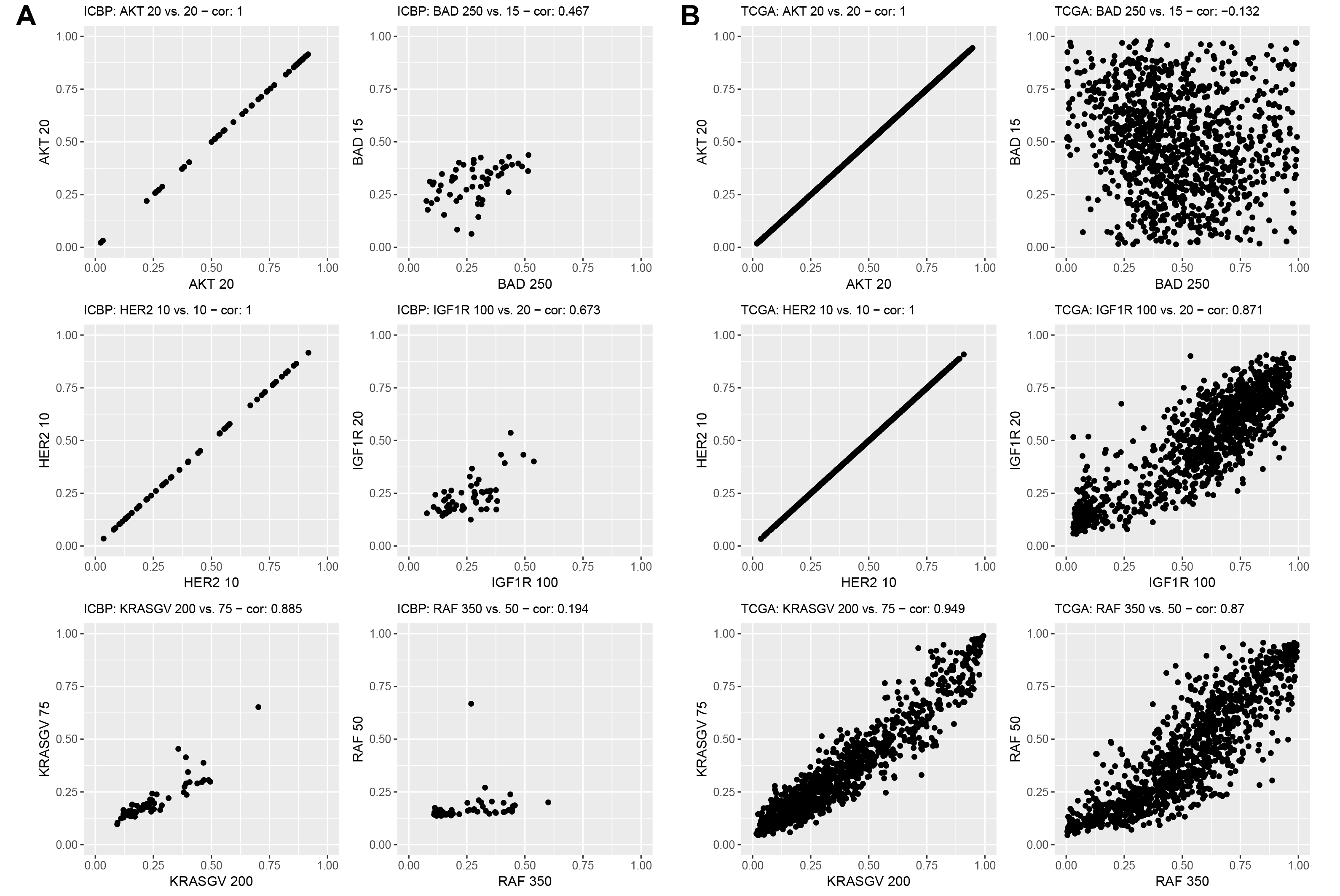

Supplement: Supplementary file 2 — Fig. S1. Reduction of RNA-sequencing based gene expression signature gene lists for NanoString panel in a) ICBP and b) TCGA for AKT1, BAD, HER2, IGF1R, KRASG12 V, and RAF1 pathways. (PNG 2175 kb) (PNG 415 kb) [file 12885_2019_6052_MOESM2_ESM.png]

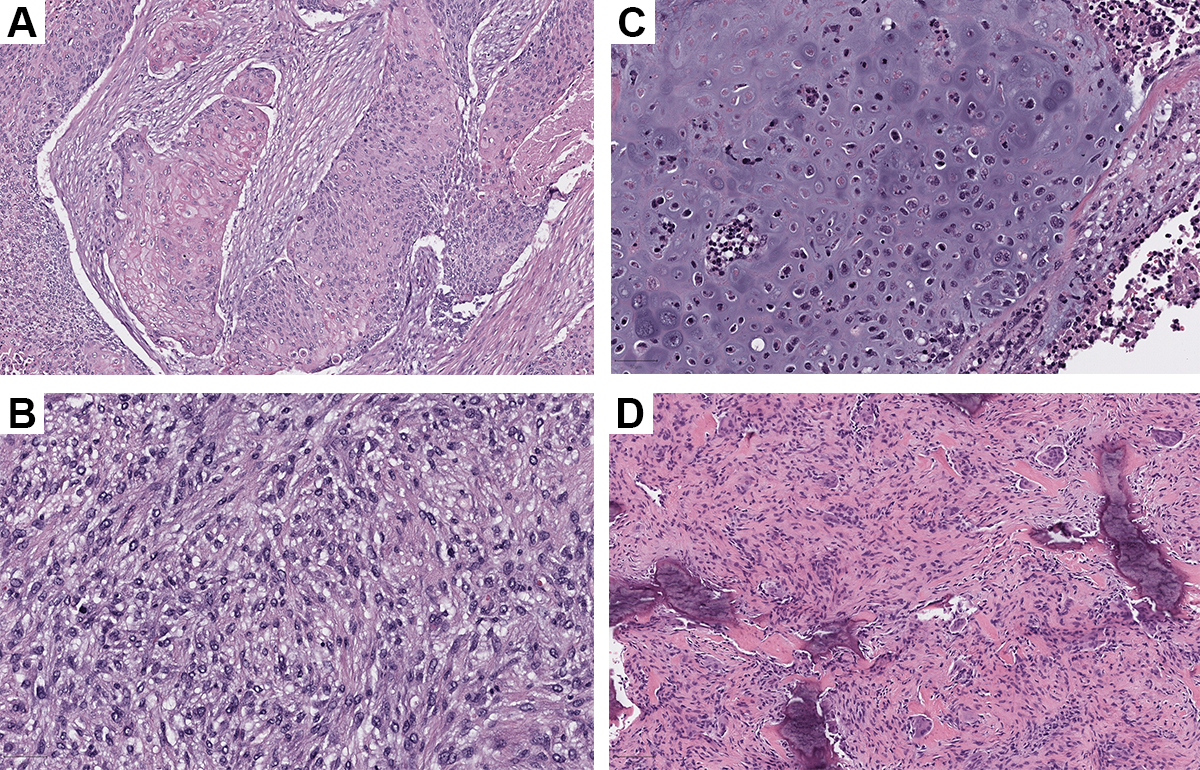

Supplement: Supplementary file 4 — Fig. S2. Representative hematoxylin and eosin slides for a) squamous b) spindle cell c) mesenchymal (chondroid) and d) mesenchymal (osteoid) metaplastic breast cancer histological subtypes. (TIF 574 kb) (PNG 2175 kb) [file 12885_2019_6052_MOESM4_ESM.png]

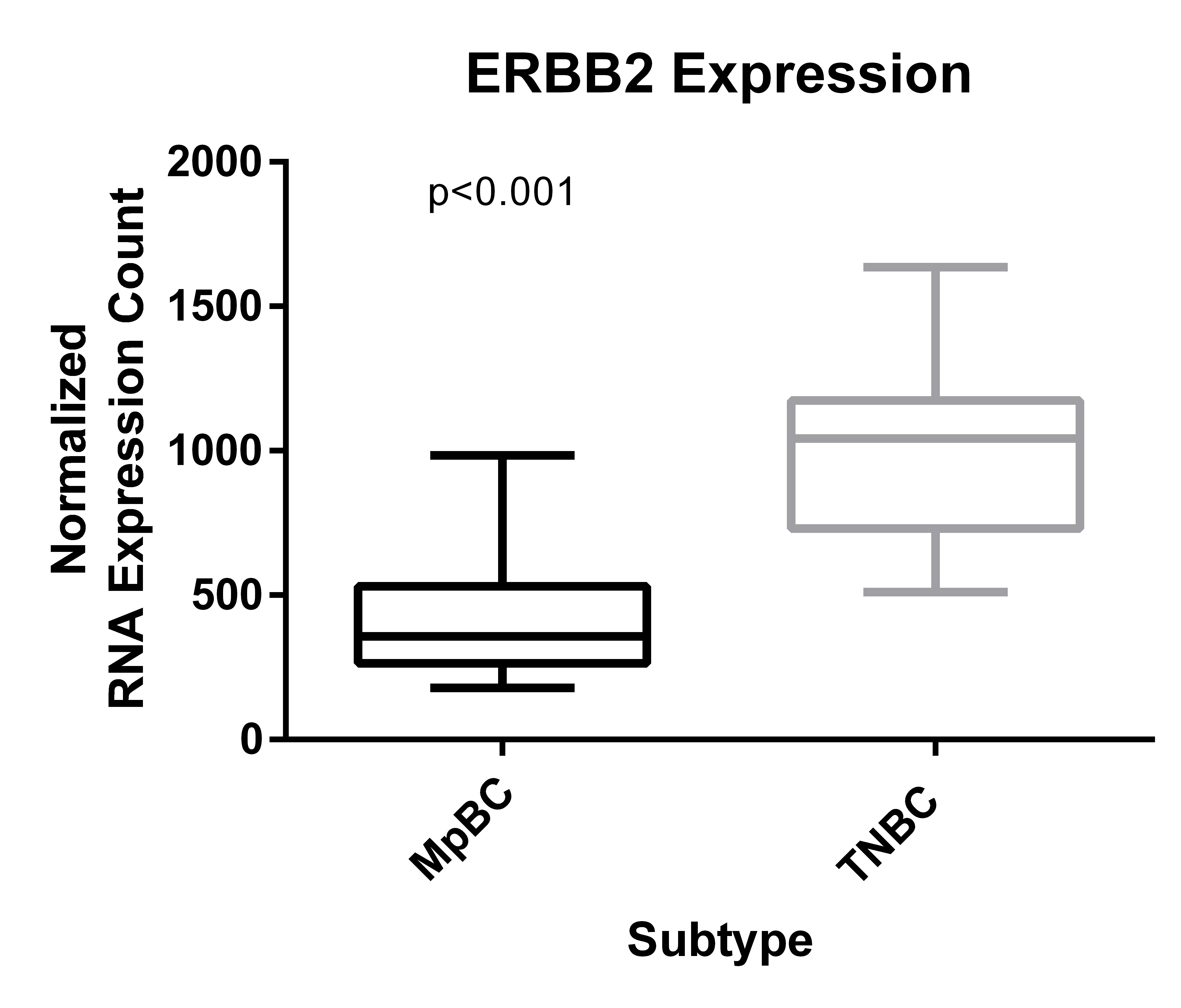

Supplement: Supplementary file 5 — Fig. S3. ERBB2/HER2 gene expression in MpBC and TNBC samples. (XLSX 19 kb) (TIF 272 kb) [file 12885_2019_6052_MOESM5_ESM.tif]
